# Surgical management of traumatic diaphragmatic hernia: a single institutional experience of more than two decades

**DOI:** 10.1186/s12893-021-01141-2

**Published:** 2021-03-19

**Authors:** Xicheng Deng, Zuosheng Deng, Erjia Huang

**Affiliations:** 1 Department of Cardiothoracic Surgery, Hunan Children’s Hospital, No. 86 Ziyuan Road, Changsha, 410007 Hunan China; 2 Department of Cardiothoracic Surgery, First People’s Hospital of Chenzhou, Chenzhou, 423000 Hunan China; 3 Department of Cardiothoracic Surgery, The Second Xiangya Hospital, Central South University, Changsha, 410011 Hunan China

**Keywords:** Traumatic diaphragmatic hernia, Surgery, CT scan, Traumatic diaphragmatic injury

## Abstract

**Background:**

We present here our experience with surgical management of traumatic diaphragmatic hernia, trying to find out the era impact of different periods on the outcome and risk factors of mortality.

**Methods:**

A series of 63 patients with traumatic diaphragmatic hernia were referred to us and operated on during March, 1990-August, 2017. The patient records were reviewed and statistically analyzed to demonstrate injury characteristics and to find out optimal treatment strategy, risk factors of death as well as the difference between two periods (1990–2005, 2005–2017) divided by introduction of computed tomography at our institution.

**Results:**

The overall mean age was 31.2 ± 16.3 years old with a female to male ratio of 11/52. The mechanism was penetrating trauma in 19 cases (30.2%), and blunt trauma in 44 cases (69.9%). Two thirds of the patients in the second group (2005–2017) yet none in the first group (1990–2005) underwent computed tomography. Ten patients (15.9%), of which 8 in the first and the other 2 in the second group (p = .042), had late diagnoses. The most commonly used incision was a thoracotomy (n = 43, 89.6%). There was no statistical difference in etiology or mortality between the two periods. Univariate analysis showed survivors were younger, and had lesser injury severity scores (ISS) and lower American Association for the Surgery of Trauma (AAST) grade than non-survivors. By multivariate logistic regression analysis, increased age (odds ratio, 1.275; p = .013) and greater ISS (OR, 1.174; p = .028) were risk factors of death in all patients.

**Conclusions:**

High-definition computed tomography has significantly improved the preoperative diagnosis rate. The transthoracic approach could be used in selected cases with traumatic diaphragmatic hernia with good outcomes. Patients with greater ISS and advanced ages are at a higher risk of death.

## Background

Diaphragmatic injuries were first described in 1541 by Sennertus [1]. This condition is not uncommon with an incidence as high as 5% for patients hospitalized after motor vehicle accidents and 15% for patients after penetrating injuries to the lower chest and upper abdomen [2–4]. And almost half of these injuries develop into hernias [5]. In view of its location between the abdomen and chest, an injured diaphragm is almost always associated with other thoracic or abdominal injuries [5] and potentially life threatening.

The advances in trauma diagnosis, critical care and surgery have greatly changed the management of diaphragmatic injuries over the last two decades. Diagnostic modalities include from traditional plain radiographs [6], ultrasonography [7], to computed tomography (CT), diagnostic laparoscopy [8–10] and thoracoscopy [11]. Moreover, surgery has evolved from open thoracotomy or laparotomy to minimally invasive endoscopy procedures. Here we conducted this study to summarize the characteristics of traumatic diaphragmatic hernia (TDH) as well as associated injuries, and to examine the effect brought by the introduction of CT. We also tried to identify predictors of death in this study.

## Methods

### Data collection

The study was approved by the Institutional Review Board of First People’s Hospital of Chenzhou and all methods were performed in accordance with the relevant guidelines and regulations. Informed consent was waived for retrospective nature of the study. The inclusion criteria were patients with TDH who underwent surgical treatment from March 1990 to August 2017 at our institution, a tertiary referral center. The diagnosis may have been made before or during surgery. If the hernia diagnosis was made during operation or not within 72 h upon admission, it was defined as a late diagnosis. Exclusion criteria: 1. patients who only received conservative management after injury; 2. acute patients (injured within 3 days) who had been surgically treated before admission to our institution and had not received a secondary operation. Specifically, the study period was divided by the year 2005 when the CT was introduced at our institution and has changed the diagnostic mode significantly. Patient records, operative reports, and discharge summaries were collected retrospectively to obtain data of patient demographics, type of injury sustained and mechanisms, operative procedures, and survival. We categorized mechanisms of injury as blunt trauma for motor vehicle collision (MVC), crush injuries from fallen walls/buildings, and penetrating traumas for gunshot wound (GSW) and stab wound (SW). Injury Severity Scores (ISS) [12] for each patient were calculated by the same person throughout (one of the authors: X.D.). All patients were analyzed according to the mechanism of injury, ISS, time point of diagnosis, associated injuries, surgical routes, including thoracotomy, laparotomy or thoracoabdominal incisions and outcomes. For the classification of diaphragmatic injury, the American Association for the Surgery of Trauma (AAST) diaphragmatic injury score [13] was used.

### Patient management and operative details

Upon admission, patients were first managed conservatively to stabilize respiratory and circulatory status. Meanwhile, workups were performed to make clear diagnoses preoperatively. If the patient was unstable or presented with a large open wound, an exploratory procedure was planned and performed as soon as possible. The majority of this series was approached via a posterolateral thoracotomy. If the injury was mainly abdominal, a laparotomy would be chosen. In cases with thoracic-abdominal injuries, a combined thoracoabdominal approach would be used to access both the chest and abdomen. Crush and firearm injuries might cause a large area of soft tissue defect after debridement, whereby a Dacron patch might be needed to alleviate tension after repair. Otherwise, we chose to close the hernia directly with pledged interrupted mattress sutures. When necessary, the ruptured edge of the diaphragm could be sutured to the intercostal muscles or around the ribs. All sutures used were silk ranging 4–7 metric. A chronic hernia usually suggested massive adhesion between hernia contents and the chest and there might be difficulty finding and repairing the diaphragm. Extensive mobilization is necessary before restoration of hernia contents and closure of ruptured diaphragm.

### Statistical analysis

Data analysis was performed using SPSS version 17.0 for Windows (IBM, Armonk, NY, USA). All quantitative data were presented as mean values ± standard error of the mean. Early mortality (during hospitalization or within 30 days following operation) was used as the endpoint. All patients were analyzed according to the mechanism of injury, Injury Severity Scores (ISS), time point of diagnosis, associated injuries, surgical routes and mortality using the Student *t* test or the Chi-square test. Multivariate logistic regression was used to determine significant risk factors of the probability of non-survival. P ≤ 0.05 was considered significant.

## Results

A total of 63 patients (52 males, 82.5%) with traumatic diaphragmatic hernia were identified in patient record documents from 1990 to 2017 (36 cases in 1990–2005 as the first group, 27 cases in 2005–2017 as the second group). None of the 63 patients was excluded. The mean age for all was 31.2 ± 16.3 years old (range, 0.5–69). Penetrating TDH occurred in 19 of 63 (30.2%) compared with 44 from blunt injuries (69.8%). Stab wounds caused 14 (73.7%) of the 19 penetrating injuries, and gunshot wounds resulted in the rest. Motor vehicle crashes resulted in 27 of 44 blunt injuries (61.4%), and crush injuries for the rest. The mean age (23.6 ± 9.5 years old) in the penetrating group was significantly younger (p = 0.042) than in the blunt group (33.5 ± 19.7 years old), while the mean ISS (p = 0. 029) and AAST grade (p = 0. 014) were significantly lower in the penetrating group than in the blunt group. Out of the 63 cases, 45 were injured in the left, 16 in the right and the rest 2 in both sides (p = 0.002) (Table 1). Patients with acute TDH usually present with significant respiratory or circulatory symptoms, such as respiratory distress from atelectasis, hemothorax or mass effect of hernia content or hypotension or even shock from major bleeding or delayed transfer. For chronic TDH, patients usually presented with abdominal pain, ileus, or vomiting.

**Table 1:** Demographics of the population

	Total, n (%)	Blunt, n (%)	Penetrating, n (%)	P value
Patients No	63	44 (69.8)	19 (30.2)	N/A
Mean age, years ± SD	31.2 ± 16.3	33.5 ± 19.7	23.6 ± 9.5	.042
Male	52 (82.5)	36	16	N/A
Mean ISS ± SD	26.6 ± 20 .7	36.3 ± 23.2	20.8 ± 17.4	.029
AAST grade	2.1 ± 0.7	2.8 ± 0.9	1.5 ± 0.3	.014
Survival to discharge	56 (88.9)	40 (63.5)	16 (25.4)	.734
Mortality	7 (11.1)	4 (9.1)	3 (15.8)	.670

*N/A* not available, *AAST* American Association for the Surgery of Trauma

There were 33 associated injuries including 3 head injuries, 8 thoracic, 11 abdominal solid organs, 5 abdominal hollow viscera, 3 pelvis and spinal. The stomach was the most frequently found organ herniating into the chest (32 of 63, 50.8%), followed by the spleen (17 of 63, 3.0%), and the small bowel (9 of 63, 14.3%). The TDH was diagnosed preoperatively in 53 of 63 patients (84.1%). Late diagnosis was made in 10 including 6 (9.5%) diagnosed during operation in the same hospital stay, and 4 (6.3%) missed initially and diagnosed 4 months to 12 years later. Two thirds of the patients (19/27) in the second group compared with none in the first group underwent CT scan. Ten patients (15.9%), of which 8 in the first group and the other 2 in the second group (p = 0.042), had late diagnoses. (Table 2).

**Table 2:** Imaging modality in different periods

	1990–2005	2005–2017	P value
Mechanisms			
Penetrating (19)/Blunt (44)	8/28	11/16	.113
Chest X-ray (63)	36	27	1.000
Ultrasound (44)	23	21	.235
Barium meal (7)	4	3	1.000
CT scan (19)	0	19	.000
Preoperative diagnosis	28/36 (77.8%)	25/27 (92.6%)	.002
Mortality	5/36	2/27	.685

The patients received 65 operations in total, one for each patient, except for two patients who had been injured bilaterally in the chest and underwent two separate procedures each. The most common operative approach was thoracotomy, in 43 cases. The rest included 11 laparotomies, 9 thoracoabdominal incisions (Table 3). Of the 63 cases, 7 died. Four died of severe multi-organ failure and 3 of delayed referral from underresourced primary treating hospital. The rest were discharged uneventfully. No significant difference in mortality between two periods was found.

**Table 3:** Incisions with etiology of blunt and penetrating diaphragmatic injuries

Causes n (%)	Thoracotomy^a^	Laparotomy	Thoracoabdominal incision
MVC 27 (42.9)	23	3	1
Crush 17 (27.0)	14	2	1
SW 14 (22.2)	5	4	5
GSW 5 (7.9)	3	1	1
Total 63 (100)	43 (68.2)	11 (17.4)	9 (14.2)

^a^: including 2 bilateral thoracotomies patients, each patient counted as one

*GSW* Gunshot wound, *MVC* motor vehicle collision, *SW* stab wound, *TDH* traumatic diaphragmatic hernia

Univariate analysis showed older age (p = 0.017), higher ISS (p = 0.026) and AAST grade (p = 0.014) were associated with non-survival (Table 4). Multivariate logistic regression analysis suggested that advanced age (p = 0.013) and higher ISS (p = 0.028) were predictors of death (Table 5).

**Table 4:** Univariate analysis for the prediction of non-survival

	Survivors (n = 56)	Non-survivors (n = 7)	P value
Mean age, years ± SD	30.2 ± 16.7	41 ± 18.8	.017
ISS ± SD	25 (11.7)	47 (17.9)	.026
Sex			1.000
Male	46	6	
Female	10	1	
AAST grade ± SD	1.4 ± 0.3	2.9 ± 0.7	.014

*ISS* Injury severity score, *SD* standard deviation, *AAST* American Association for the Surgery of Trauma

**Table 5:** Multivariate logistic regression analysis for the prediction of non-survival

	OR	95% CI	P value
Sex	0.983	0.264–2.541	0.891
Mechanism	0.639	0.131–1.567	0.233
Age	1.275	1.023–1.196	.013
ISS	1.174	0.968–1.105	.028

*ISS* Injury severity score, *OR* Odds Ratio

## Discussion

Our results show that the majority of the cases were caused by blunt injuries. Older mean age, higher ISS score and AAST grade in blunt group were significantly different than in penetrating group. The age difference implicates a socioeconomic impact on the mechanisms of injury. Higher ISS score and AAST grade seem to suggest more severe injuries from blunt mechanism. However, different mechanism did not translate to difference in mortality in multivariate analysis. A number of series showed more patients with traumatic diaphragmatic injury (TDI) suffered from penetrating trauma as compared with blunt trauma [5, 14] while other series have suggested that the majority of TDI, up to 80%, resulted from blunt trauma [8, 15–18]. Lewis [5] thought that these discrepancies in frequency are likely the result of variations in geographic and socioeconomic factors of the study populations. In our TDH cohort, blunt trauma was predominantly more frequent. Presumably, this is because we had selectively excluded the cases of TDI without herniation. We speculate that since a lot of TDI were not able to be recognized promptly, especially those with penetrating injuries without typical herniation. These cases might have been missed in some previous studies. And it may also have added to the discrepancies in frequency of injury mechanisms. The most common causes of TDH in our series (MVC, GSW, and SW) are consistent with previously reported data. [8, 14, 17, 19, 20] MVC comprised 61.4% of all cases, which was by far the most common cause. All [5], or at least a large proportion [17], of penetrating injuries were due to stab and GSWs, which was consistent with our study.

It is suggested that diaphragmatic injuries be diagnosed before the complications like diaphragmatic hernia and strangulation occur since mortality and morbidity increase after the herniation and strangulation of the abdominal viscera in the thoracic cavity. However, according to our experience, in a significant proportion of the cases diaphragmatic injuries were insidious with no visible signs on Chest X-ray, which was reported to be very useful and with sensitivities up to 94% in the presence of a hernia [21]. In the early period, we utilized conventional chest X-ray, abdominal ultrasound and barium meal trying to achieve early diagnoses. Though all cases enrolled in the study presented with herniation, as reported previously [22], some of them were not diagnosed in a timely fashion. The most common incidence of delayed diagnosis was the right-sided diaphragmatic rupture [23]. According to reports, only 17% of the cases of right-sided rupture are diagnosed with chest X-ray [24]. In our study, 6 (60%) cases of delayed diagnosis were of right-sided hernia. Another important reason probably is the films were interpreted by surgeons. Although they were able to identify all cases of diaphragmatic hernia, many cases of injury without a typical hernia were missed [5, 25, 26]. Furthermore, sometimes the hernia might have been mimicking an elevated diaphragm or it might have been misdiagnosed for hemothorax or encapsulated effusion especially in the right hemithorax with the liver herniating into the chest. As mentioned above, there were 4 delayed diagnoses which were confirmed 4 months to 12 years later after discharge from hospital. Delayed or missed diagnoses may place patients at risk for morbidity and mortality [27–29]. In some cases, it could be insidious for delayed hernia formation or small openings [30, 31]. In the recent period, we have been using CT scan (initially, 16 multi-slice, then 64 multi-slice) characterized by high space definition and multi-planer reconstruction to help diagnose and have achieved a minimal number of missed diaphragmatic injuries. CT has greatly enhanced the possibility of early diagnosis and is reported to have a sensitivity of 71% (78% for the left and 50% for the right) and a specificity of 100% and an accuracy of 88% for the left and 70% for the right-sided injuries. [32] With CT scan, the early diagnosis rates have become significantly higher than it was before (p = 0.042) (Table 2).

The incidence of associated injuries was 52.4%, and the most common associated injury was rib fracture followed by intra-abdominal injuries, which were similar to previous series [18, 33]. Some studies have suggested a nearly 100% incidence of associated injuries, different from ours. This may be explained by the different enrollment criteria. In our study, all patients died before admission were excluded while some previous studies included such patients. Since associated injuries add to ISS scores, suggesting higher mortality, those who died before hospitalization probably had higher ratio of associated injuries.

The operative approach of choice is affected by whether there are associated injuries. Though it was reported that the incidence of associated additional intra-abdominal injuries was up to 100% of patients [17, 34–36], we chose a thoracotomy as our main approach (68.2%) (Table 3) for an incidence of 25.4% (16/63) for associated abdominal injuries of in our series. In our experience, a posterolateral thoracotomy warrants a good exposure of diaphragm as well as part of the abdominal organs. We have found it easier to reduce the herniated contents and to repair the diaphragm through a thoracotomy when there are no intra-abdominal injuries [37]. Importantly, in cases of delayed presentation, thoracotomy is an accepted approach as it is difficult to release the intra thoracic adhesions through a laparotomy [38]. Many authors choose laparotomy or laparoscopy as the route of choice [8–10, 39]. A Laparotomy may be used in patients presenting with abdominal symptom or physical signs and will be more frequently used in our future practice as more evidence coming up in favor of a laparotomy. In patients with severe injury in the junction between thorax and abdomen, a thoracoabdominal incision may be the approach of choice. Another important factor impacting surgeon’s choice is the familiarity and comfort level of an approach [40], which is also the case in this study. Surgeons should keep in mind that injuries might happen in both sides [30, 41]. A case in our cohort presented with shortness of breath and low saturation of blood oxygen after surgical repair of left diaphragm injury. Further investigations including x-ray and ultrasound suggested right diaphragmatic rupture and hepatic herniation into the chest. In the early stage, the liver had not herniated yet, so it had not been identified before the initial operation. The patient had to undergo another right thoracotomy and recovered without major morbidity.

The mortality in our study (11.1%) was much lower than that of other published series [14, 20, 42]. Several reasons may have contributed to it. First, our study only enrolled the patients who had survived to hospital admission, yet many had died before admission. Second, the referrals from other hospitals were more than often limited to relatively less severely injured patients, so a lot of severely or critically injured patient were not included. Third, our study focused specifically on TDH, which is only a subset of TDI. However, we found risk factors for death similar to published studies [14, 36, 43]. Older age (p = 0.017), higher ISS (p = 0.026) and AAST grade (p = 0.014) by univariate analysis were associated with non-survival. Multivariate logistic regression analysis suggested that advanced age (p = 0.013) and higher ISS (p = 0.028) were predictors of death. Same as previous report [5], no difference in mortality was found between the blunt and penetrating mechanisms. Since the volume of cohort is small, large-scale study is warranted. Importantly, we did not identify any difference in mortality between the two eras though the early diagnosis rate was higher in the second group owing to the induction of CT. We speculate that delay in transfer resulted in high mortality of severely injured patients, which did not change much between the two groups. Presumably, patients successfully transferred to our institution had lower ISS score.

It is noticeable that since the study period is very long, during which the medical and surgical management of TDH have involved significantly, the study does not aim at a punctual compare between the eras as there are plenty of confounding factors. However, it is safe to say that new imaging modality like CT has changed diagnostic strategies for this population and may potentially help improve the outcomes. Currently, we would perform a CT whenever possible when an emergency case with thoracoabdominal injury presents. And we have started using minimally invasive techniques in selected patients. A multi-disciplinary team including thoracic, general, trauma surgeons, and intensivists are being established. Hopefully, this new teamwork paradigm would result in better outcomes.

## Limitations

This study has some limitations. It was a retrospective case series study with an arbitrarily set dividing timepoint for the two eras. As mentioned before, the clinical paradigm for diagnosis and management has changed significantly over this long study period, implicating confounding factors when analyzing results. Furthermore, this limited number of patients in our series precluded sophisticated statistical analysis. There were some cases who had not been managed initially at out institution but presented later for chronic hernia, whereby recall bias may have occurred. The exclusion of patients died before admission may have caused selection bias. 

## Conclusion

Our study found that the demographics and constituent ratio of the mechanism varied between TDH and TDI. Diaphragmatic hernias were more common after blunt injury chest. Right-sided rupture was a common cause of delayed diagnosis. The application of multi-slice CT scan has significantly increased early diagnosis rate of TDH. But at present, we have not found significant difference in mortality between the two groups though significant advances in trauma management, critical care, and interventional radiology might have improved survival in the latter era. Diaphragmatic hernia repair could be done through a thoracotomy with acceptable results provided there are no intra-abdominal injuries or diaphragmatic injuries presenting late. Older age, higher ISS and AAST grade were predictors of death.

## Data Availability

The datasets used and/or analyzed during the current study available from the corresponding author on reasonable request.

## Abbreviations

ISS: Injury severity scores
AAST: American Association for the Surgery of Trauma
TDH: Traumatic diaphragmatic hernia
GSW: Gunshot wound
MVC: Motor vehicle collision
TDI: Traumatic diaphragmatic injury
CT: Computed tomography
